# Age-Dependent Heterogeneity of Murine Olfactory Bulb Astrocytes

**DOI:** 10.3389/fnagi.2020.00172

**Published:** 2020-06-09

**Authors:** Marcel Klein, Christian Lohr, Damian Droste

**Affiliations:** Division of Neurophysiology, University of Hamburg, Hamburg, Germany

**Keywords:** astrocyte, olfactory bulb, cell morphology, heterogeneity, aging, Sholl analysis

## Abstract

Astrocytes have a high impact on the structure of the central nervous system, as they control neural activity, development, and plasticity. Heterogeneity of astrocytes has been shown before, but so far only a few studies have demonstrated heterogeneous morphology of astrocytes concerning aging. In this study, we examined morphologic differences of astrocyte subpopulations in adult mice and the progression of these differences with age. We surveyed astrocytes in olfactory bulb slices of mice aged 3 months, 1 year and 2 years (three animals each age group), based on their appearance in anti-GFAP immunostaining. Based on this data we established three different types of astrocytes: type I (stellate), type II (elliptic), and type III (squid-like). We found that with the advanced age of the mice, astrocytes grow in size and complexity. Major changes occurred between the ages of 3 months and 1 year, while between 1 and 2 years no significant development in cell size and complexity could be detected. Our results show that astrocytes in the olfactory bulb are heterogeneous and undergo morphological transformation until late adolescence but not upon senescence. Structural plasticity is further substantiated by the expression of vimentin in some astrocyte processes in all age groups.

## Introduction

Astrocytes are amongst the most prominent cell types in the central nervous system. They fulfill a plethora of functions and are increasingly considered as an important part of the central nervous system not only for homeostasis but also for the function of neurons (Allen and Barres, 2009; Khakh and Sofroniew, 2015; Verkhratsky and Nedergaard, 2018; Kastanenka et al., 2020). Participation in the blood-brain barrier, transportation of nutrients from blood vessels to neurons and linking neuronal activity to vascular responses (neurovascular coupling) is among the most acknowledged properties of astrocytes (Barros and Deitmer, 2010; Lohr et al., 2014; Chatton et al., 2016; Liebner et al., 2018). Furthermore, astrocytic processes reach into the vicinity of neuronal synapses, a concept known as the tripartite synapse (Araque et al., 1999), where they detect neuronal neurotransmitter release, take up transmitters to prevent neurotoxicity, and even secrete their own neurotransmitters known as gliotransmitters to adjust synaptic transmission (Rothstein et al., 1994; Deitmer et al., 1998; Volterra and Meldolesi, 2005; Perea and Araque, 2007; Verkhratsky and Nedergaard, 2018). It has been shown that astrocytes can modulate neuronal activity and synaptogenesis (Chung et al., 2013, 2016; Diniz et al., 2014b, 2016; Liddelow et al., 2017).

Morphological diversity and heterogeneity of astrocytes have been observed since as early as the 19th century by *Camillo Golgi and Ramón y Cajal* (Garcia-Lopez et al., 2010; Oberheim et al., 2012). In the first attempt of classification, astrocytes were categorized as protoplasmatic astrocytes in the gray matter and fibrillary astrocytes located in the white matter of the brain (Barres, 2008; Köhler et al., 2019). More recent studies show that astrocytes are highly diverse depending on the brain region, microenvironment, and developmental stage (Barres, 2008). Several studies have shown that astrocytes display heterogeneous molecular and functional properties (Miller, 2018; Köhler et al., 2019; Matias et al., 2019). In detail, different populations of astrocytes express different sets of receptors, are of different developmental origin and differ in morphology, physiology and metabolism (Lerea and McCarthy, 1989; Shinoda et al., 1989; Zhang and Barres, 2010; Oberheim et al., 2012; Droste et al., 2017; Mölders et al., 2018; Ziemens et al., 2019). Furthermore, astrocytes vary in the impact they have on their environment such as in their positive or negative guidance of axonal growth (Smith et al., 1986; Rudge and Silver, 1990) or their influence on neuronal differentiation, circuit formation and synaptogenesis (Denis-Donini et al., 1984; Emsley and Macklis, 2006; Oberheim et al., 2012; Diniz et al., 2014a,b; Buosi et al., 2018). Experimental evidence has also shown that these different types of astrocytes can show different Ca^2+^ dynamics, electrophysiological properties, and gap-junction formation (Zhang and Barres, 2010). Molofsky and Deneen (2015) postulated that morphological differences are strongly linked to functional diversity among astrocytes. This diversity in form and function is very likely to have an impact on the three-dimensional structure and organization of the central nervous system (Zhang and Barres, 2010). Another interesting aspect of astrocytic heterogeneity is its development during aging. It has been shown that astrocyte reactivity is not only a sign of the onset of neurodegenerative diseases but also a normal development with increasing age in rodents, non-human and human primates (Nichols et al., 1993; Robillard et al., 2016; Rodríguez-Arellano et al., 2016; Gómez-Gonzalo et al., 2017; Gomez-Arboledas et al., 2018). In the aging hippocampus and frontal cortex, astrocyte reactivity is observed mainly in areas where synaptic loss and age-related cognitive decline occur (Rodríguez-Arellano et al., 2016). In the hippocampal dentate gyrus and CA1 region, astrocytes have been shown to develop age-related hypertrophy represented by a reorganization of GFAP, increasing surface, total volume, and somata volume of astrocytes in aged mice (Rodríguez et al., 2014). Furthermore, age-dependent inflammation or “inflammaging” is an important risk factor in aged brains (Colombo and Farina, 2016; Palmer and Ousman, 2018). Astrocytes isolated from an aged mouse brain have shown an increased inflammatory phenotype (Orre et al., 2014). Generally, these studies show that astrocytes undergo age-related changes in morphology and gene expression that may influence brain function with advanced age (Boisvert et al., 2018; Clarke et al., 2018). So far, age-dependent heterogeneity of astrocytes in the olfactory bulb has not been studied in detail. The olfactory bulb possesses some unique properties. It is the only special sense that lack primary efferent projections to the thalamus since it contains its own intrinsic olfactory thalamus and comprises direct projections to the olfactory cortex (Kay and Sherman, 2007; Sarnat et al., 2017; Rotermund et al., 2019). Also, the olfactory bulb is an exceptional brain region because adult-born neurons derived from the subventricular zone migrate in large numbers into the olfactory bulb and are implemented into existing neural circuits throughout life (Pignatelli and Belluzzi, 2010; Sakamoto et al., 2014). The question arises whether, in this quasi-juvenile environment, that might act as a “fountain of youth,” aging astrocytes show the same signs of hypertrophy found in other brain regions or remain in a rather juvenile state.

In the present study, we investigated morphological changes of olfactory bulb astrocytes during a time range between three and 24 months. We identified three different types of astrocytes based on their morphology: stellate, elliptic, and unidirectional “squid-like” astrocytes. All types of astrocytes increased in size and complexity between the age of 3 and 12 months, while only minor morphological changes were found between 12 and 24 months.

## Methods

### Animals and Preparation

We used C57BL/6 mice of both genders at the age of 3 months, 1 year, and 2 years for immunostaining. For each age group, three animals were used. Mice were provided by V. Sakk and H. Geiger (Ulm, Germany), sacrificed and their heads fixed in paraformaldehyde solution (4% PFA in 0.1 mM PBS, see below). Animal dissection was performed according to the European Union’s and local animal welfare guidelines (Behörde für Gesundheit und Verbraucherschutz, Hamburg, Germany).

### Immunohistology and Image Acquisition

Fixed whole mouse brains were washed in phosphate-buffered saline (PBS: 130 mM NaCl; 7 mM Na_2_HPO_4_; 3 mM NaH_2_PO_4_; pH adjusted to 7.4 with NaOH). After dissection, the olfactory bulbs were stored in 4% PFA solution at 4°C. Olfactory bulb slices (sagittal, 150 μm) were prepared using a vibratome (VT1000, Leica Benzheim Germany). Afterwards, slices were incubated in blocking solution [5% normal goat serum (NGS); 0.5% Triton X100; in PBS] for 1 h. Subsequently, slices were incubated with the primary antibodies anti-GFAP (chicken, polyclonal, 1:500; #ab4674, Abcam, Cambridge, UK), vimentin (rabbit, polyclonal, 1:200; #GTX100619, GeneTex, Irvine, CA, USA) and anti-S100 (rabbit, polyclonal, 1:1,000; #Z0311, Dako, Hamburg, Germany) for 36–48 h at 4°C. The anti-S100 antibody strongly detects S100B, an abundant glial protein, and weakly S100A1 that is found in neurons (Ilg et al., 1996). Antibodies were diluted in 0.5% NGS; 0.05% Triton X100 in PBS. Slices were then incubated with the secondary antibodies (goat anti-rabbit, Alexa Fluor 488, 1:1,000, Invitrogen Thermo Fisher, Darmstadt, Germany, and goat anti-chicken, Alexa Fluor 555, 1:1,000, Abcam, Cambridge, UK) and for nuclear staining with 2 μM Hoechst 33342 (Molecular Probes, Eugine, USA) in PBS for another 24 h at 4°C. Slices were mounted using a self-hardening embedding medium (Immu-Mount, Thermo Fisher). We used a confocal microscope (C1 Eclipse, Nikon, Düsseldorf, Germany) to acquire image stacks using a 40×/NA 1.3 oil immersion lens. The *z* axial step size was 150 nm.

### Classification of Astrocytes

**Figure 1:**
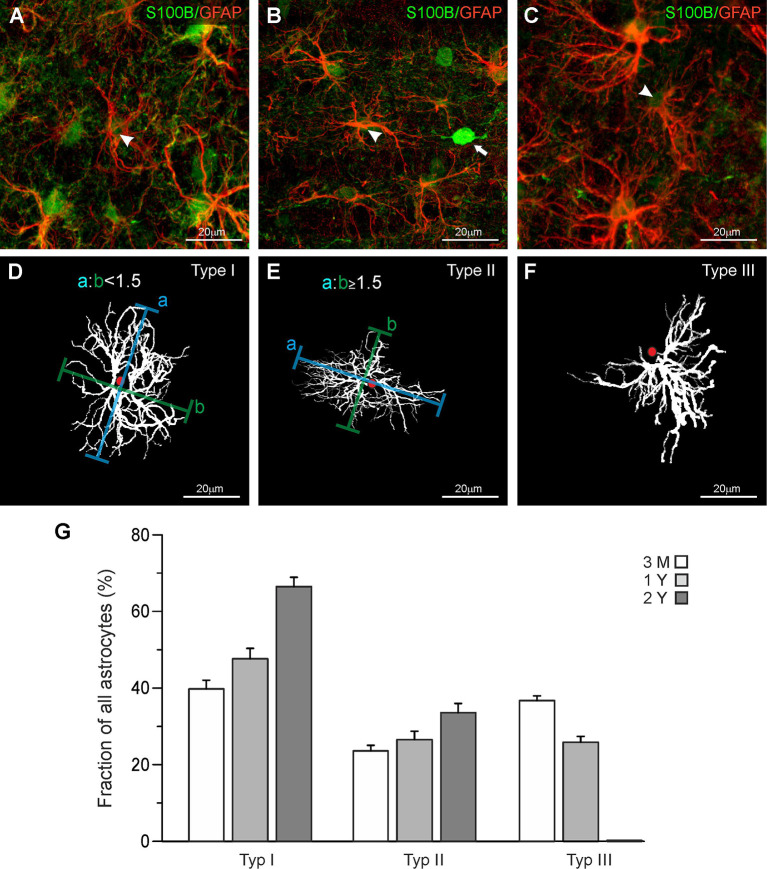
Classification of astrocytes. **(A–C)** Anti-GFAP (red), and anti-S100B staining (green) of olfactory bulb astrocytes. Arrowheads point to astrocytes that have been traced and are depicted in panels **(D–F)**. **(D)** Astrocytes with a ratio of longitudinal-to-orthogonal extension of less than 1.5 were defined as stellate type I astrocytes. **(E)** Astrocytes with a ratio of longitudinal-to-orthogonal extension of 1.5 and more were defined as elliptic type II astrocytes. **(F)** Astrocytes with nearly all processes oriented unidirectionally were defined as “squid-like” type III astrocytes. **(G)** The fraction of type I astrocytes strongly increased between the age of 3 months and 2 years, while the fraction of type II astrocytes only weakly increased. Type III astrocytes decreased in number between 3 months and 1 year and disappeared thereafter.

Maximum intensity projections were made using Fiji ImageJ (Schindelin et al., 2012) and adjusted for brightness and contrast. We established a set of parameters to categorize astrocytes regarding the orientation and distribution of cell processes. This led to an identification of three different types of astrocytes (Figure 1). The first type of astrocytes, type I or “stellate” astrocyte, is defined by a regular distribution of processes around the soma. The distance from the center of the soma to the outer limits of the astrocytes, processes are similar for each orientation, giving the astrocyte a circular appearance (Figures 1A,D). Type II or “elliptic” astrocytes show increased growth of processes from two opposed poles of the soma (Figures 1B,E). As a definition, an astrocyte was categorized elliptic when the ratio of the longest dimension and the orthogonal dimension was larger 1.5. The “squid-like” type III astrocytes develop more than 90% of their processes in one direction, originating from one central area on the soma (Figures 1C,F). The processes reaching out from the astrocytes give the astrocyte a shape that resembles a squid.

### Sholl Analysis

**Figure 2:**
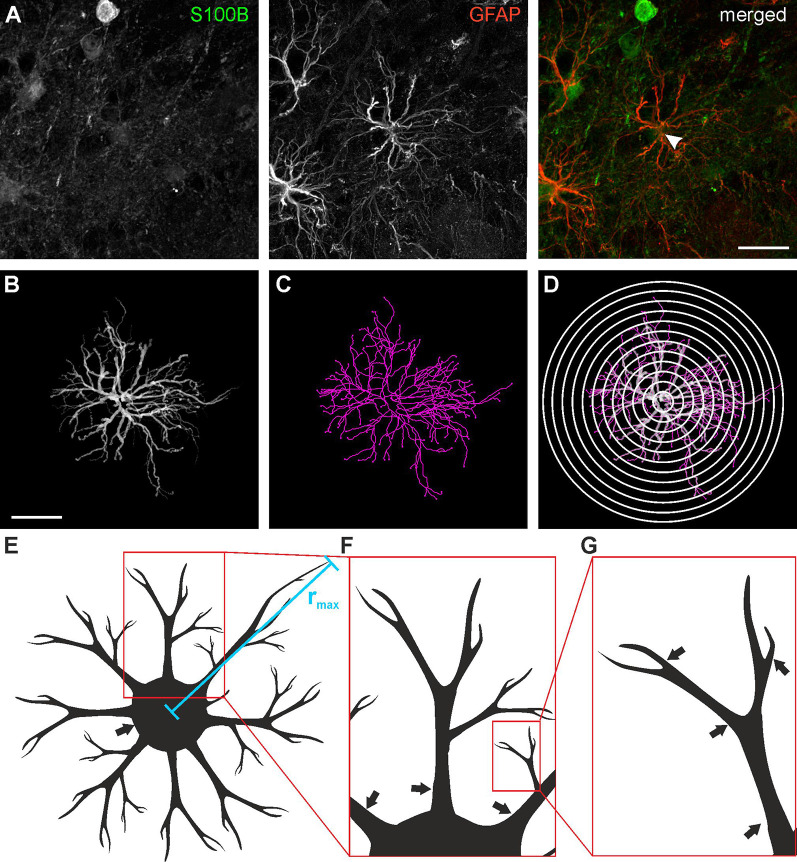
Astrocyte tracing and Sholl analysis. **(A)** Anti-GFAP (red) and anti-S100B staining (green) of a type I astrocyte (arrowhead). **(B)** Three-dimensional tracing of the astrocyte depicted in panel **(A)**. **(C)** Filament plot of the traced astrocyte. **(D)** Sholl plot with 4 μm interval. **(E)** Schematic drawing of astrocyte morphology analysis for the maximum radius (*r*_max_), **(F)** central processes (arrows) and **(G)** branch points (arrows). Scale bars: 20 μm.

Astrocytes were traced in three dimensions using the GFAP staining data sets and the Simple Neurite Tracer plug-in of ImageJ following the protocol of Tavares et al. (2017), yielding a skeleton of the astrocyte processes (Figure 2). The location of the soma was added to the traced image data set using the S100B staining. Based on the traces derived from Simple Neurite Tracer, a three-dimensional Sholl analysis was performed using a distance of 4 μm between the concentric spheres (Figure 2D). The data acquired with ImageJ was imported to Excel 2010 (Microsoft) and the following parameters were analyzed: Total and relative number of processes, the maximum radius as well as the number and length of the processes originating from the soma of each astrocyte (Figures 2E,F) was determined. A central process is defined by its origin in the soma (Figure 2F; arrows indicate central processes), each branch point creates an additional process. The total number of processes is the sum of all individual processes (Figure 2G; arrows indicate processes).

### Statistical Analysis

All statistical analyses were performed using IBM SPSS Statistics 23. For each astrocyte type, 15 cells equally distributed over the olfactory bulb layers were traced from three mice for each age group. For each age group, we used the Kolmogorov–Smirnov-Lilliefors-Test to confirm normal distribution, a univariate variance analysis test, and a student’s *t*-test to test for significance. Means were considered statistically different when the error probability *P* was < 0.05.

## Results

### Different Types of Astrocytes in the Olfactory Bulb

In the present study, we investigated mouse astrocytes in the external plexiform layer and deeper layers of the olfactory bulb, while we omitted astrocytes in the glomerular layer. Astrocytes in the glomerular layer of mouse olfactory bulbs are well studied and it has been shown that they represent a unique population in terms of morphology and physiology (Doengi et al., 2009; Roux et al., 2011; Beiersdorfer et al., 2019, 2020). Comparing the morphological appearance of olfactory bulb astrocytes we distinguished three groups of astrocytes that differ in their morphological features. Figure 1 shows each of the three astrocyte types we defined as seen in the GFAP staining (Figures 1A–C) and as a projection image created with Simple Neurite Tracer in ImageJ (Figures 1D–F). GFAP-positive astrocytes always colocalized with S100 staining that was used to define the soma. Interestingly, we also found S100-positive cell bodies that lack GFAP labeling and possess clear neuron-like shape (Figure 1B; white arrow). This might be due to the reactivity of the antibody with neuronal S100A1, while the immunostaining in astrocytes results from binding to S100B (Ilg et al., 1996). Also, some neurons such as deep cerebellar neurons have been reported to express S100B (Vives et al., 2003) and we cannot exclude the expression of S100B in some neurons of the olfactory bulb. GFAP-positive astrocytes with somata in the direct vicinity of these S100-expressing neurons were not chosen for analysis to avoid any misinterpretation. The astrocyte in Figure 1D shows a regular distribution of its processes around the soma and hence was defined as a “stellate” type I astrocyte. Figure 1E shows an astrocyte with an elongated structure and an elliptic orientation of its processes. The outline of this astrocyte shows that its horizontal diameter is at least 1.5 times as long as its vertical diameter. Thus, this astrocyte was categorized as an “elliptic” type II astrocyte. Figure 1F shows an astrocyte that has virtually all its processes oriented in one direction originating in a specific area of the soma. The structure resembles the shape of a squid, which is why we defined this type of astrocyte as “squid-like” type III astrocytes. Interestingly, type III astrocytes with somata in the internal plexiform layer often projected their processes into the mitral cell layer between the cell bodies of the mitral cells (Supplementary Figure S1). However, type III astrocytes were also found in the external plexiform layer and the granule cell layer.

### Age-Related Distribution of Astrocyte Types in the Olfactory Bulb Layers

Structural and functional changes of astrocytes have been implicated in aging and astrocyte dysfunction is considered to contribute to cognitive impairment in age-related neurodegenerative diseases such as Alzheimer’s disease (Rodríguez-Arellano et al., 2016; Verkhratsky et al., 2019). Therefore, we were interested in whether the distribution of the three types of astrocytes changes in the course of aging and compared the fraction of type I, type II, and Type III astrocytes in the olfactory bulb of mice of different ages. We analyzed 349 astrocytes at an age of 3 months, 343 astrocytes at 1 year, and 244 astrocytes at an age of 2 years, using three mice for each age group. Since there were only minor differences in the distribution of the three types of astrocytes between the layers, data of all layers investigated were pooled (Supplementary Figure S2). In total, the relative number of stellate (type I) astrocytes increased from 39.7 ± 2.3% at an age of 3 months to 47.6 ± 2.7% and 66.4 ± 2.4% at an age of 1 and 2 years, respectively (Figure 1G). The fraction of elliptic (type II) astrocytes increases slightly from 23.6 ± 1.5% at 3 months to 26.5 ± 2.2% at one and 33.6 ± 2.4% at 2 years. Type III astrocytes, in contrast, decreased in number between the age of 3 months (36.7 ± 1.3%) and 1 year (25.9 ± 1.6%) and entirely disappeared after 2 years. Using the size of the area of interest and the depth of each z step of the confocal image, we determined the volume of each image stack so we could calculate the relative density of astrocytes in the olfactory bulb. The total density of astrocytes decreases slightly but not significantly after the age of 3 months (0.032 astrocytes/1,000 μm^3^ at 3 months, 0.026 astrocytes/1,000 μm^3^ at 1 year and 0.028 astrocytes/1,000 μm^3^ at 2 years).

### Sholl Analysis of Olfactory Bulb Astrocytes

To evaluate the development of the different types of astrocytes with age in more detail, we performed a Sholl analysis to quantify morphological and structural changes with increasing age in each astrocyte type (Figure 2). For all the following analyses, 15 astrocytes were investigated for each cell subtype and each age group, resulting in a total of 120 astrocytes. The Sholl analysis provides information on the number of cell processes at a certain distance from the center of the cell, i.e., the soma. It also gives the maximum radius of the cell (Figure 2E).

**Figure 3:**
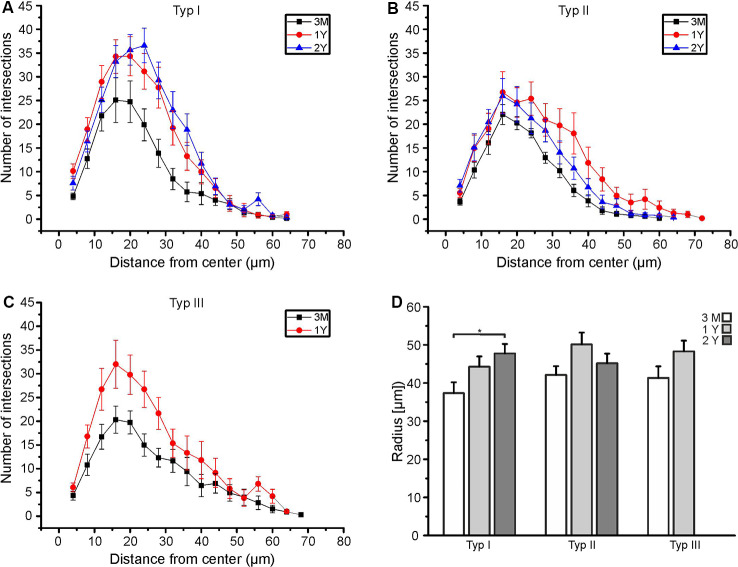
Age-dependent Sholl analysis of astrocytes. **(A)** Sholl plot of type I astrocytes, **(B)** type II astrocytes and **(C)** type III astrocytes at an age of 3 months (black), 1 year (red), and 2 years (blue). **(D)** Averaged maximum radii of all astrocytes analyzed. Type I astrocytes significantly increased in the maximum radius between 3 months and 2 years (*p < 0.05).

We analyzed type I, type II, and type III astrocytes and compared the data of animals at an age of 3 months, 1 year and 2 years. At an age of 3 months, the maximal number of intersections (peak of the Sholl plot) was at an average distance of 16 μm to the cell soma for all types of astrocytes (Figures 3A–C). Between the age of 3 months and 1 year, the total number of intersections increased, in particular at distances of 16 μm and larger. Hence, the peak of the Sholl plots increased in amplitude and shifted to larger distances, most obviously that of type I and type II astrocytes (Figures 3A,B). There was no further increase in the number of intersections (and hence cell processes) between the ages of 1 year and 2 years. Type III astrocytes increased the number of their processes more evenly along with the entire range of distances from the soma between 3 months and 1 year (Figure 3C). Since type III astrocytes disappeared between 1 year and 2 years, no data for 2-year-old mice are shown in Figure 3C. In addition to the increased number of cell processes, the maximum radius of the astrocytes increased upon aging (Figure 3D). The radius of type I astrocytes was 37.3 ± 2.9 μm at the age of 3 months and 44.3 ± 2.7 μm at 1 year. It increases significantly to 47.7 ± 2.5 μm at the age of 2 years. The radius of type II and type III astrocytes also increased between 3 months and 1–2 years, however, the differences between ages did not reach the level of significance. The decrease in astrocyte density and the increase in astrocyte size suggests that the increase in the volume of the olfactory bulbs between the age of 3 months and 1 year is compensated by the growth of astrocyte processes rather than the generation of new astrocytes to allow for coverage of the entire parenchyma with astrocyte processes.

### Astrocytes Gain Complexity With Increasing Age

**Figure 4:**
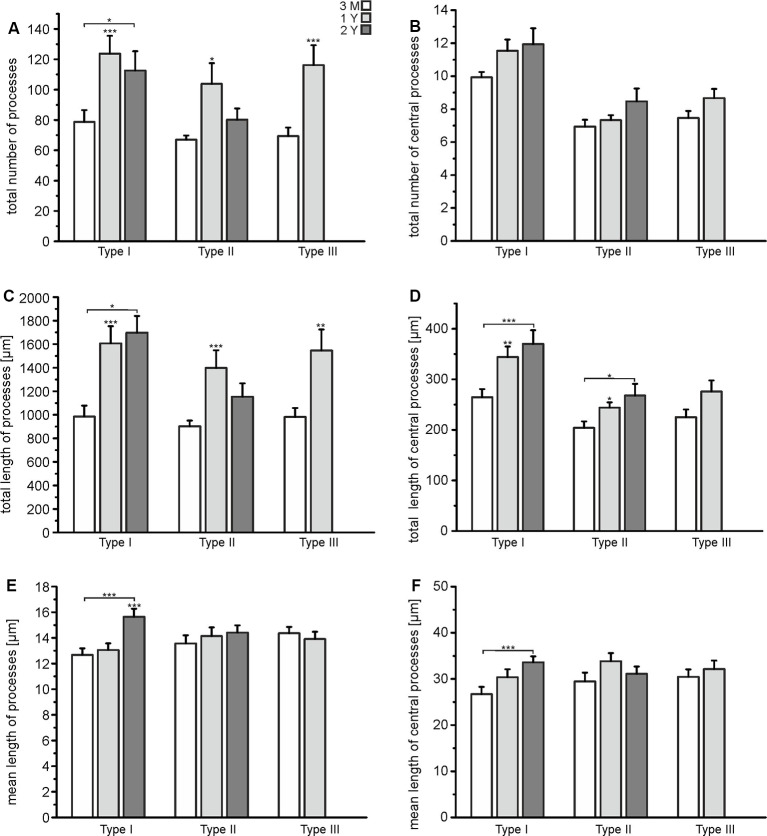
Quantification of astrocyte complexity. **(A)** The total number of processes of each type of astrocyte for each age group. **(B)** The total number of central processes of each type of astrocyte for each age group. **(C)** The total length of all astrocytes of each type of astrocyte for each age group. **(D)** The total length of all central processes of each type of astrocyte for each age group. **(E)** Mean length of all processes for each type of astrocyte and each age group. **(F)** Mean length of central processes for each type of astrocyte and each age group. *p < 0.05; **p < 0.01; ***p < 0.001.

The results of the Sholl analysis suggest that astrocytes might gain complexity with age as the number of intersections and hence branching points increased. We performed a more elaborate analysis of the tracing data to find out in detail which parameters of cell morphology change upon aging. We calculated the total number of cell processes, where any cell process is defined by its endpoint, i.e., a process that branches once gives rise to two processes (see Figure 2G). Exact values are summarized in Supplementary Table S1. The number of processes of all types of astrocytes increased significantly between the age of 3 months and 1 year, while there was no further increase between 1 year and 2 years (Figure 4A). However, the number of central processes, i.e., processes that originate in the soma of the astrocyte, did not increase significantly upon aging (Figure 4B). This is in line with the data of the Sholl plots showing an increase in the number of intersections preferentially at distant processes, but not close to the soma (Figure 3). The total length of cell processes (i.e., the sum of the lengths of all processes of an astrocyte) significantly increased upon aging, regardless whether all processes or only central processes were considered (Figures 4C,D), confirming that astrocytes did not only increase the number of processes but also their overall size (radius). The increase in total length could be due to an increased length of all single processes that belong to an astrocyte, or an increased number of processes, as suggested by the results shown in Figures 4A,B. The mean length of all processes and central processes did not change significantly upon aging, indicating that the increase in the total length of the cell processes was indeed caused by the increased number of cell processes (Figures 4E,F). As an exception, type I astrocytes significantly increased the mean length of their processes between the age of 3 months and 2 years. Our results show that all types of astrocytes in the olfactory bulb increased their complexity, in particular the number of branch points, cell processes, and the overall size, between the ages of 3 months and 1 year, whereas between 1 and 2 years most changes in morphological parameters were neglectable.

### Vimentin Immunoreactivity in Astrocyte Processes

**Figure 5:**
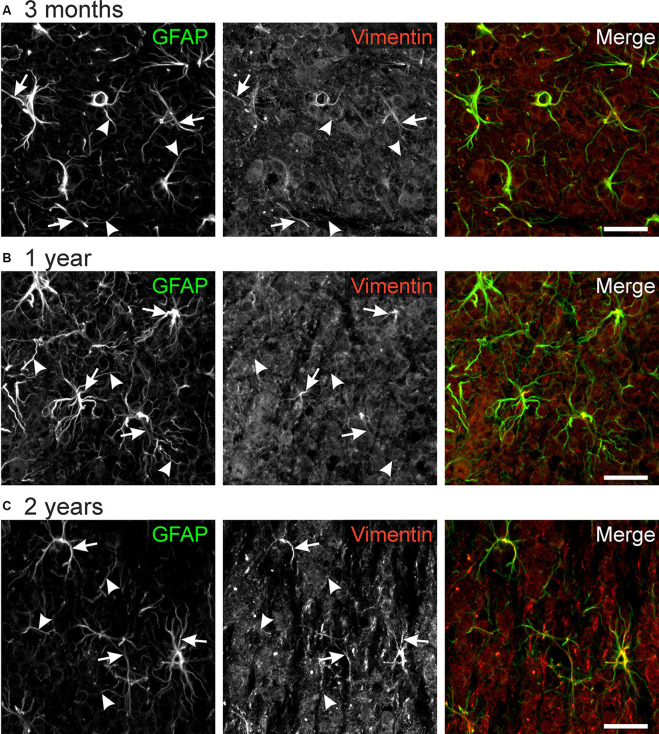
GFAP-vimentin colocalization in the mouse olfactory bulb. Anti-GFAP (green) and anti-vimentin (red) immunoreactivity colocalizes in some astrocyte processes (arrows), while most GFAP-positive processes were vimentin-negative (arrowheads). This result was observed in mice of the age of 3 months **(A)**, 1 year **(B)**, and 2 years **(C)**. Scale bar: 20 μm.

Vimentin, an intermediate filament related to GFAP, is highly expressed in immature astrocytes. Its expression is downregulated upon maturation but is upregulated in mature astrocytes after an injury that requires structural plasticity such as trauma and stroke (Pekny, 2001; Lewis and Fisher, 2003). Hence, vimentin is considered as a marker for astrocyte plasticity. We found immunoreactivity against vimentin in some but not all astrocyte processes (Figure 5). No differences between different ages could be observed, suggesting that astrocytes in the olfactory bulb retain some capability of structural plasticity throughout life. Hence, vimentin-positive astrocyte processes might account for the structural changes we found in our study such as the rearrangement of cell processes from a squid-like to a stellate morphology.

## Discussion

The results of this study show that astrocytes of the olfactory bulb build morphologically heterogeneous subpopulations. These differences led us to categorize olfactory bulb astrocytes into three morphological types. Additionally, we found that these astrocytes undergo morphological changes with the increasing age of the animal.

### Different Types of Astrocytes in the Rodent Olfactory Bulb

It has been recognized for a long time that astrocytes throughout the CNS show a large diversity regarding their morphology, physiology, and thus their function (Zhang and Barres, 2010; Oberheim et al., 2012). In the present study, we have shown that astrocytes in deeper layers of the mouse olfactory bulb show morphological differences and can be categorized into three groups depending on the orientation of their cell processes concerning the cell body. In a study on rat olfactory bulb astrocytes, Bailey and Shipley (1993) defined six subtypes of astrocytes sorting them by shape and location. However, three subtypes were only found in the superficial nerve layer and a glomerular layer which we omitted since they are already well characterized (Roux et al., 2011; Beiersdorfer et al., 2019, 2020; Fischer et al., 2020). The remaining three astrocyte subtypes they described were confirmed by our findings in the murine olfactory bulb; Bailey and Shipley (1993) described circular (stellate type I) astrocytes, elongated (elliptic type II) astrocytes and semicircular astrocytes which project their processes only in one direction concerning the soma and hence resemble the “squid-like” astrocyte (type III) described in our results. The majority of astrocytes in the EPL, IPL, and GCL of the rat olfactory bulb belonged to the stellate and “squid-like” astrocytes (Bailey and Shipley, 1993), similar to the results we found in the mouse olfactory bulb at an age of 3 months. Unfortunately, in the study on rat olfactory bulb the age of the animals is not given, however, it is very unlikely that they were older than a few months since the study did not address aging (Bailey and Shipley, 1993). However, another study addressing postnatal development of the rat olfactory bulb also found circular (stellate), semicircular/elongated (elliptic), and wedge-shaped (“squid-like) astrocytes (Chiu and Greer, 1996). Astrocytes in the olfactory bulb not only differ in morphology but also in physiology. While all olfactory bulb astrocytes respond to ATP and ADP with Ca^2+^ signaling, only subpopulations respond to dopamine, GABA, and glutamate (Doengi et al., 2008, 2009; Droste et al., 2017; Fischer et al., 2020).

### Distribution and Development of Astrocyte Types Depending on Age

While astrocyte heterogeneity has been discussed for a while now, only a few studies exist that investigate the matter concerning aging. A recent review brought astrocyte heterogeneity in connection with aging and age-related disease, highlighting the importance of that topic (Matias et al., 2019). In the present study, we focused on the heterogeneity of the olfactory bulb astrocytes concerning aging. We employed the Sholl analysis that not only provides information about the size of the cells but also about the number and complexity of the cell branches. We analyzed three-dimensional data sets that covered the largest part of the cell branches. However, it should be noted that astrocyte processes were visualized by staining for GFAP, an intermediate filament that is mainly found in the larger cell processes of mature astrocytes while omitting fine processes such as perisynaptic microdomains. The larger, GFAP-positive cell processes increased in length and complexity between the age of 3 months and 1 year, while only minor changes, if any, were found between the age of 1 and 2 years. Although the olfactory bulb is functionally mature at an age of 3 months, it further increases in size for a short time, while at an age of 1 year, the final size is reached (Hinds and McNelly, 1977; Pomeroy et al., 1990). Therefore, the increase in size and complexity of olfactory bulb astrocytes may reflect an adaptation to the increase in the size of the olfactory bulb. This is confirmed by the apparent decrease in astrocyte density with age, suggesting that the total number of astrocytes remains constant despite the increase in olfactory bulb volume, which requires an increased length of the cell processes to occupy the entire olfactory bulb tissue. Between the ages of 1 and 2 years, in contrast, little changes in morphological complexity occurred. The only major change is the entire disappearance of the “squid-like” astrocytes with a simultaneous and equivalent increase in cell number of stellate astrocytes until the age of 2 years. This suggests a rearrangement of the cell processes of “squid-like” astrocytes to yield stellate astrocytes. Structural redistribution is further indicated by relatively high expression of vimentin in some astrocyte processes since vimentin is a marker for immature astrocytes and astrocyte reactivity associated with structural plasticity (Pekny, 2001; Bramanti et al., 2010). In the rat olfactory bulb, vimentin is strongly expressed embryonically and during the first postnatal days in GFAP-negative radial glial cells (Bailey et al., 1999). During postnatal development, radial glia converts into mature astrocytes, vimentin expression is down-regulated and GFAP expression is up-regulated (Chiu and Greer, 1996; Bailey et al., 1999). The change in expression of intermediate filaments and morphology of glial cells accompanies the embryonic and early postnatal formation of layers, dendritic growth and synaptogenesis in the olfactory bulb both in rodents and in humans (Bailey et al., 1999; Sarnat and Yu, 2016).

The lack of significant changes in structural complexity of mature olfactory bulb astrocytes between the age of 1 and 2 years is in contrast to aging astrocytes in the hippocampus, where hypertrophic astrocytes evolve between ages of 3 months and 2 years, while astrocytes in the entorhinal cortex decrease in size during the same time range (Rodríguez et al., 2014). Viola et al. (2009) reported that astrocytes in the hippocampus CA1 region react to an enriched environment with a morphological transition. These astrocytes, in particular those oriented along with Schaffer collaterals, tend to adopt a stellate shape and develop more and longer central processes, comparable to type I astrocytes we defined in the olfactory bulb. In the hippocampal dentate gyrus, in contrast, the complexity of astrocytes decreased upon aging, and this decrease was more profound in mice raised in an enriched environment (Diniz et al., 2016). Unique types of astrocytes are also found in other brain regions. Bergmann glial cells, specialized astrocytes in the cerebellar cortex, retain the radial orientation of embryonic radial glial cells and serve as scaffold guiding the postnatal migration of immature granule cells (Bluhm et al., 2015). Müller glial cells in the retina are elongated bipolar astrocytes that span the entire retina. This unique structural property renders Müller cells ideal to serve as optical fiber and guide light from the vitreous body to the outer nuclear layer comprising photoreceptor cells (Lohr et al., 2014).

## Conclusion

Our data shows that the number and total length of processes, as well as the maximal radius of astrocytes in the olfactory bulb, increases up to the age of 1 year, while the mean length of processes remains constant. This indicates the development of new short processes and astrocytes are likely in a state of high variability. Hence, astrocytes increase size and complexity to cope with the increase in olfactory bulb volume up to the age of 1 year. Besides, the persistent neurogenesis in the olfactory bulb throughout the organisms’ life might also be contributing to a high level of astrocytic plasticity at this age (Molofsky and Deneen, 2015).

## Data Availability Statement

The datasets generated for this study are available on request to the corresponding author.

## Ethics Statement

The animal study was reviewed and approved by Behörde für Gesundheit und Verbraucherschutz, Hamburg, Germany.

## Author Contributions

MK, CL, and DD designed the study. MK performed and analyzed the experiments. DD wrote the manuscript. All authors edited the manuscript.

## Conflict of Interest

The authors declare that the research was conducted in the absence of any commercial or financial relationships that could be construed as a potential conflict of interest.

## References

[B1] Allen N. J. Barres B. A. (2009). Neuroscience: glia—more than just brain glue. Nature 457, 675–677. 10.1038/457675a 19194443

[B2] Araque A. Parpura V. Sanzgiri R. P. Haydon P. G. (1999). Tripartite synapses: glia, the unacknowledged partner. Trends Neurosci. 22, 208–215. 10.1016/s0166-2236(98)01349-6 10322493

[B4] Bailey M. S. Puche A. C. Shipley M. T. (1999). Development of the olfactory bulb: evidence for glia-neuron interactions in glomerular formation. J. Comp. Neurol. 415, 423–448. 10.1002/(sici)1096-9861(19991227)415:4<423::aid-cne2>3.0.co;2-g 10570454

[B3] Bailey M. S. Shipley M. T. (1993). Astrocyte subtypes in the rat olfactory bulb: morphological heterogeneity and differential laminar distribution. J. Comp. Neurol. 328, 501–526. 10.1002/cne.903280405 8429132

[B5] Barres B. A. (2008). The mystery and magic of glia: a perspective on their roles in health and disease. Neuron 60, 430–440. 10.1016/j.neuron.2008.10.013 18995817

[B6] Barros L. F. Deitmer J. W. (2010). Glucose and lactate supply to the synapse. Brain Res. Rev. 63, 149–159. 10.1016/j.brainresrev.2009.10.002 19879896

[B7] Beiersdorfer A. Scheller A. Kirchhoff F. Lohr C. (2019). Panglial gap junctions between astrocytes and olfactory ensheathing cells mediate transmission of Ca^2+^ transients and neurovascular coupling. Glia 67, 1385–1400. 10.1002/glia.23613 30883940

[B8] Beiersdorfer A. Wolburg H. Grawe J. Scheller A. Kirchhoff F. Lohr C. (2020). Sublamina-specific organization of the blood brain barrier in the mouse olfactory nerve layer. Glia 68, 631–645. 10.1002/glia.23744 31696993

[B9] Bluhm B. Laffer B. Hirnet D. Rothermundt M. Ambree O. Lohr C. (2015). Normal cerebellar development in S100B-deficient mice. Cerebellum 14, 119–127. 10.1007/s12311-014-0606-z 25342137

[B10] Boisvert M. M. Erikson G. A. Shokhirev M. N. Allen N. J. (2018). The aging astrocyte transcriptome from multiple regions of the mouse brain. Cell Rep. 22, 269–285. 10.1016/j.celrep.2017.12.039 29298427 PMC5783200

[B11] Bramanti V. Tomassoni D. Avitabile M. Amenta F. Avola R. (2010). Biomarkers of glial cell proliferation and differentiation in culture. Front. Biosci. 2, 558–570. 10.2741/s85 20036968

[B12] Buosi A. S. Matias I. Araujo A. P. B. Batista C. Gomes F. C. A. (2018). Heterogeneity in synaptogenic profile of astrocytes from different brain regions. Mol. Neurobiol. 55, 751–762. 10.1007/s12035-016-0343-z 28050794

[B13] Chatton J. Y. Magistretti P. J. Barros L. F. (2016). Sodium signaling and astrocyte energy metabolism. Glia 64, 1667–1676. 10.1002/glia.22971 27027636

[B14] Chiu K. Greer C. A. (1996). Immunocytochemical analyses of astrocyte development in the olfactory bulb. Dev. Brain Res. 95, 28–37. 10.1016/0165-3806(96)00055-7 8873973

[B15] Chung W. S. Clarke L. E. Wang G. X. Stafford B. K. Sher A. Chakraborty C.. (2013). Astrocytes mediate synapse elimination through MEGF10 and MERTK pathways. Nature 504, 394–400. 10.1038/nature12776 24270812 PMC3969024

[B16] Chung W. S. Verghese P. B. Chakraborty C. Joung J. Hyman B. T. Ulrich J. D.. (2016). Novel allele-dependent role for APOE in controlling the rate of synapse pruning by astrocytes. Proc. Natl. Acad. Sci. U S A 113, 10186–10191. 10.1073/pnas.1609896113 27559087 PMC5018780

[B17] Clarke L. E. Liddelow S. A. Chakraborty C. Munch A. E. Heiman M. Barres B. A. (2018). Normal aging induces A1-like astrocyte reactivity. Proc. Natl. Acad. Sci. U S A 115, E1896–E1905. 10.1073/pnas.1800165115 29437957 PMC5828643

[B18] Colombo E. Farina C. (2016). Astrocytes: key regulators of neuroinflammation. Trends Immunol. 37, 608–620. 10.1016/j.it.2016.06.006 27443914

[B19] Deitmer J. W. Verkhratsky A. J. Lohr C. (1998). Calcium signalling in glial cells. Cell Calcium 24, 405–416. 10.1016/s0143-4160(98)90063-x 10091009

[B20] Denis-Donini S. Glowinski J. Prochiantz A. (1984). Glial heterogeneity may define the three-dimensional shape of mouse mesencephalic dopaminergic neurones. Nature 307, 641–643. 10.1038/307641a0 6694754

[B21] Diniz D. G. de Oliveira M. A. de Lima C. M. Fôro C. A. Sosthenes M. C. Bento-Torres J.. (2016). Age, environment, object recognition and morphological diversity of GFAP-immunolabeled astrocytes. Behav. Brain Funct. 12:28. 10.1186/s12993-016-0111-2 27719674 PMC5056502

[B22] Diniz L. P. Matias I. C. Garcia M. N. Gomes F. C. (2014a). Astrocytic control of neural circuit formation: highlights on TGF-β signaling. Neurochem. Int. 78, 18–27. 10.1016/j.neuint.2014.07.008 25125369

[B23] Diniz L. P. Tortelli V. Garcia M. N. Araújo A. P. Melo H. M. Silva G. S.. (2014b). Astrocyte transforming growth factor β 1 promotes inhibitory synapse formation *via* CaM kinase II signaling. Glia 62, 1917–1931. 10.1002/glia.22713 25042347

[B24] Doengi M. Deitmer J. W. Lohr C. (2008). New evidence for purinergic signaling in the olfactory bulb: A_2A_ and P2Y_1_ receptors mediate intracellular calcium release in astrocytes. FASEB J. 22, 2368–2378. 10.1096/fj.07-101782 18310463

[B25] Doengi M. Hirnet D. Coulon P. Pape H. C. Deitmer J. W. Lohr C. (2009). GABA uptake-dependent Ca^2+^ signaling in developing olfactory bulb astrocytes. Proc. Natl. Acad. Sci. U S A 106, 17570–17575. 10.1073/pnas.0809513106 19805126 PMC2765163

[B26] Droste D. Seifert G. Seddar L. Jädtke O. Steinhäuser C. Lohr C. (2017). Ca^2+^-permeable AMPA receptors in mouse olfactory bulb astrocytes. Sci. Rep. 7:44817. 10.1038/srep44817 28322255 PMC5359673

[B27] Emsley J. G. Macklis J. D. (2006). Astroglial heterogeneity closely reflects the neuronal-defined anatomy of the adult murine CNS. Neuron Glia Biol. 2, 175–186. 10.1017/s1740925x06000202 17356684 PMC1820889

[B28] Fischer T. Scheffler P. Lohr C. (2020). Dopamine-induced calcium signaling in olfactory bulb astrocytes. Sci. Rep. 10:631. 10.1038/s41598-020-57462-4 31959788 PMC6971274

[B29] Garcia-Lopez P. Garcia-Marin V. Freire M. (2010). The histological slides and drawings of cajal. Front. Neuroanat. 4:9. 10.3389/neuro.05.009.2010 20339483 PMC2845060

[B30] Gomez-Arboledas A. Davila J. C. Sanchez-Mejias E. Navarro V. Nuñez-Diaz C. Sanchez-Varo R.. (2018). Phagocytic clearance of presynaptic dystrophies by reactive astrocytes in Alzheimer’s disease. Glia 66, 637–653. 10.1002/glia.23270 29178139 PMC5814816

[B31] Gómez-Gonzalo M. Martin-Fernandez M. Martínez-Murillo R. Mederos S. Hernández-Vivanco A. Jamison S.. (2017). Neuron-astrocyte signaling is preserved in the aging brain. Glia 65, 569–580. 10.1002/glia.23112 28130845 PMC5314210

[B32] Hinds J. W. McNelly N. A. (1977). Aging of the rat olfactory bulb: growth and atrophy of constituent layers and changes in size and number of mitral cells. J. Comp. Neurol. 72, 345–367. 10.1002/cne.901710304 833360

[B300] Ilg E. C. Schäfer B. W. Heizmann C. W. (1996). Expression pattern of S100 calcium-binding proteins in human tumors. Int. J. Cancer 68, 325–332. 10.1002/(SICI)1097-0215(19961104)68:3<325::AID-IJC10>3.0.CO;2-7 8903474

[B33] Kastanenka K. V. Moreno-Bote R. De Pittà M. Perea G. Eraso-Pichot A. Masgrau R.. (2020). A roadmap to integrate astrocytes into Systems Neuroscience. Glia 68, 5–26. 10.1002/glia.23632 31058383 PMC6832773

[B301] Kay L. M. Sherman S. M. (2007). An argument for an olfactory thalamus. Trends Neurosci. 30, 47–53. 10.1016/j.tins.2006.11.007 17161473

[B34] Khakh B. S. Sofroniew M. V. (2015). Diversity of astrocyte functions and phenotypes in neural circuits. Nat. Neurosci. 18, 942–952. 10.1038/nn.4043 26108722 PMC5258184

[B35] Köhler S. Winkler U. Hirrlinger J. (2019). Heterogeneity of astrocytes in grey and white matter. Neurochem. Res. [Epub ahead of print]. 10.1007/s11064-019-02926-x 31797158

[B36] Lerea L. S. McCarthy K. D. (1989). Astroglial cells *in vitro* are heterogeneous with respect to expression of the α 1-adrenergic receptor. Glia 2, 135–147. 10.1002/glia.440020302 2568341

[B37] Lewis G. P. Fisher S. K. (2003). Up-regulation of glial fibrillary acidic protein in response to retinal injury: its potential role in glial remodeling and a comparison to vimentin expression. Int. Rev. Cytol. 230, 263–290. 10.1016/s0074-7696(03)30005-1 14692684

[B38] Liddelow S. A. Guttenplan K. A. Clarke L. E. Bennett F. C. Bohlen C. J. Schirmer L.. (2017). Neurotoxic reactive astrocytes are induced by activated microglia. Nature 541, 481–487. 10.1038/nature21029 28099414 PMC5404890

[B39] Liebner S. Dijkhuizen R. M. Reiss Y. Plate K. H. Agalliu D. Constantin G. (2018). Functional morphology of the blood-brain barrier in health and disease. Acta Neuropathol. 135, 311–336. 10.1007/s00401-018-1815-1 29411111 PMC6781630

[B40] Lohr C. Grosche A. Reichenbach A. Hirnet D. (2014). Purinergic neuron-glia interactions in sensory systems. Pflugers Arch. 466, 1859–1872. 10.1007/s00424-014-1510-6 24705940

[B41] Matias I. Morgado J. Gomes F. C. A. (2019). Astrocyte heterogeneity: impact to brain aging and disease. Front. Aging Neurosci. 11:59. 10.3389/fnagi.2019.00059 30941031 PMC6433753

[B42] Miller S. J. (2018). Astrocyte heterogeneity in the adult central nervous system. Front. Cell. Neurosci. 12:401. 10.3389/fncel.2018.00401 30524236 PMC6262303

[B43] Mölders A. Koch A. Menke R. Klöcker N. (2018). Heterogeneity of the astrocytic AMPA-receptor transcriptome. Glia 66, 2604–2616. 10.1002/glia.23514 30370555

[B44] Molofsky A. V. Deneen B. (2015). Astrocyte development: a guide for the perplexed. Glia 63, 1320–1329. 10.1002/glia.22836 25963996

[B45] Nichols N. R. Day J. R. Laping N. J. Johnson S. A. Finch C. E. (1993). GFAP mRNA increases with age in rat and human brain. Neurobiol. Aging 14, 421–429. 10.1016/0197-4580(93)90100-p 8247224

[B46] Oberheim N. A. Goldman S. A. Nedergaard M. (2012). Heterogeneity of astrocytic form and function. Methods Mol. Biol. 814, 23–45. 10.1007/978-1-61779-452-0_3 22144298 PMC3506190

[B47] Orre M. Kamphuis W. Osborn L. M. Melief J. Kooijman L. Huitinga I.. (2014). Acute isolation and transcriptome characterization of cortical astrocytes and microglia from young and aged mice. Neurobiol. Aging 35, 1–14. 10.1016/j.neurobiolaging.2013.07.008 23954174

[B48] Palmer A. L. Ousman S. S. (2018). Astrocytes and Aging. Front. Aging Neurosci. 10:337. 10.3389/fnagi.2018.00337 30416441 PMC6212515

[B49] Pekny M. (2001). Astrocytic intermediate filaments: lessons from GFAP and vimentin knock-out mice. Prog. Brain Res. 132, 23–30. 10.1016/s0079-6123(01)32062-9 11544992

[B50] Perea G. Araque A. (2007). Astrocytes potentiate transmitter release at single hippocampal synapses. Science 317, 1083–1086. 10.1126/science.1144640 17717185

[B51] Pignatelli A. Belluzzi O. (2010). “Neurogenesis in the adult olfactory bulb,” in The Neurobiology of Olfaction, ed. Menini A. (Boca Raton, FL: CRC Press), 267–303. 21882421

[B52] Pomeroy S. L. LaMantia A. S. Purves D. (1990). Postnatal construction of neural circuitry in the mouse olfactory bulb. J. Neurosci. 10, 1952–1966. 10.1523/JNEUROSCI.10-06-01952.1990 2355260 PMC6570307

[B53] Robillard K. N. Lee K. M. Chiu K. B. MacLean A. G. (2016). Glial cell morphological and density changes through the lifespan of rhesus macaques. Brain Behav. Immun. 55, 60–69. 10.1016/j.bbi.2016.01.006 26851132 PMC4899176

[B54] Rodríguez J. J. Yeh C. Y. Terzieva S. Olabarria M. Kulijewicz-Nawrot M. Verkhratsky A. (2014). Complex and region-specific changes in astroglial markers in the aging brain. Neurobiol. Aging 35, 15–23. 10.1016/j.neurobiolaging.2013.07.002 23969179

[B55] Rodríguez-Arellano J. J. Parpura V. Zorec R. Verkhratsky A. (2016). Astrocytes in physiological aging and Alzheimer’s disease. Neuroscience 323, 170–182. 10.1016/j.neuroscience.2015.01.007 25595973

[B56] Rotermund N. Schulz K. Hirnet D. Lohr C. (2019). Purinergic signaling in the vertebrate olfactory system. Front. Cell. Neurosci. 13:112. 10.3389/fncel.2019.00112 31057369 PMC6477478

[B57] Rothstein J. D. Martin L. Levey A. I. Dykes-Hoberg M. Jin L. Wu D.. (1994). Localization of neuronal and glial glutamate transporters. Neuron 13, 713–725. 10.1016/0896-6273(94)90038-8 7917301

[B58] Roux L. Benchenane K. Rothstein J. D. Bonvento G. Giaume C. (2011). Plasticity of astroglial networks in olfactory glomeruli. Proc. Natl. Acad. Sci. U S A 108, 18442–18446. 10.1073/pnas.1107386108 21997206 PMC3214998

[B59] Rudge J. S. Silver J. (1990). Inhibition of neurite outgrowth on astroglial scars *in vitro*. J. Neurosci. 10, 3594–3603. 10.1523/JNEUROSCI.10-11-03594.1990 2230948 PMC6570102

[B60] Sakamoto M. Kageyama R. Imayoshi I. (2014). The functional significance of newly born neurons integrated into olfactory bulb circuits. Front. Neurosci. 8:121. 10.3389/fnins.2014.00121 24904263 PMC4033306

[B61] Sarnat H. B. Flores-Sarnat L. Wei X. C. (2017). Olfactory development, part 1: function, from fetal perception to adult wine-tasting. J. Child Neurol. 32, 566–578. 10.1177/0883073817690867 28424010

[B62] Sarnat H. B. Yu W. (2016). Maturation and dysgenesis of the human olfactory bulb. Brain Pathol. 26, 301–318. 10.1111/bpa.12275 26096058 PMC8028954

[B63] Schindelin J. Arganda-Carreras I. Frise E. Kaynig V. Longair M. Pietzsch T.. (2012). Fiji: an open-source platform for biological-image analysis. Nat. Methods 9, 676–682. 10.1038/nmeth.2019 22743772 PMC3855844

[B64] Shinoda H. Marini A. M. Cosi C. Schwartz J. P. (1989). Brain region and gene specificity of neuropeptide gene expression in cultured astrocytes. Science 245, 415–417. 10.1126/science.2569236 2569236

[B65] Smith G. M. Miller R. H. Silver J. (1986). Changing role of forebrain astrocytes during development, regenerative failure, and induced regeneration upon transplantation. J. Comp. Neurol. 251, 23–43. 10.1002/cne.902510103 3760257

[B66] Tavares G. Martins M. Correia J. S. Sardinha V. M. Guerra-Gomes S. das Neves S. P.. (2017). Employing an open-source tool to assess astrocyte tridimensional structure. Brain Struct. Funct. 222, 1989–1999. 10.1007/s00429-016-1316-8 27696155 PMC5406431

[B67] Verkhratsky A. Nedergaard M. (2018). Physiology of astroglia. Physiol. Rev. 98, 239–389. 10.1152/physrev.00042.2016 29351512 PMC6050349

[B68] Verkhratsky A. Rodrigues J. J. Pivoriunas A. Zorec R. Semyanov A. (2019). Astroglial atrophy in Alzheimer’s disease. Pflugers Arch. 471, 1247–1261. 10.1007/s00424-019-02310-2 31520182

[B69] Viola G. G. Rodrigues L. Americo J. C. Hansel G. Vargas R. S. Biasibetti R.. (2009). Morphological changes in hippocampal astrocytes induced by environmental enrichment in mice. Brain Res. 1274, 47–54. 10.1016/j.brainres.2009.04.007 19374889

[B302] Vives V. Alonso G. Solal A. C. Joubert D. Legraverend C. (2003). Visualization of S100B-positive neurons and glia in the central nervous system of EGFP transgenic mice. J. Comp. Neurol. 457, 404–419. 10.1002/cne.10552 12561079

[B70] Volterra A. Meldolesi J. (2005). Astrocytes, from brain glue to communication elements: the revolution continues. Nat. Rev. Neurosci. 6, 626–640. 10.1038/nrn1722 16025096

[B71] Zhang Y. Barres B. A. (2010). Astrocyte heterogeneity: an underappreciated topic in neurobiology. Curr. Opin. Neurobiol. 20, 588–594. 10.1016/j.conb.2010.06.005 20655735

[B72] Ziemens D. Oschmann F. Gerkau N. J. Rose C. R. (2019). Heterogeneity of activity-induced sodium transients between astrocytes of the mouse hippocampus and neocortex: mechanisms and consequences. J. Neurosci. 39, 2620–2634. 10.1523/JNEUROSCI.2029-18.2019 30737311 PMC6445992

