# Supplementary material for: Age-Dependent Heterogeneity of Murine Olfactory Bulb Astrocytes
Source: Front Aging Neurosci. 2020 Jun 9;12:172. doi: 10.3389/fnagi.2020.00172 (PMC7296154; doi:10.3389/fnagi.2020.00172)
Supplement: Supplementary file 1 [file Data_Sheet_1.PDF]

Supplementary Material

1 Supplementary Table 1

Numerical values of bar graphs depicted in figure 4.

Fig. 4A: Total number of processes			
	3 months	1 year	2 years
Type I	78.7 ± 7.8	123.7 ± 11.8	112.6 ± 12.7
Type II	67.0 ±2.8	103.9 ± 13.6	80.0 ± 8.1
Type III	69.3 ± 5.7	116.1 ± 13.2	-
Fig. 4B: Total number of central processes			
	3 months	1 year	2 years
Type I	9.9 ± 0.3	11.5 ± 0.7	12.0 ± 1.0
Type II	6.9 ± 0.4	7.3 ± 0.3	8.5 ± 0.8
Type III	7.5 ± 0.4	8.7 ± 0.6	-
Fig. 4C: Total length of processes [µm]			
	3 months	1 year	2 years
Type I	985.9 ± 92.9	1607.4 ± 145.7	1698.3 ± 141.1
Type II	901.9 ± 49.1	1398.8 ± 148.7	1078.4 ± 114.1
Type III	982.6 ± 75.4	1546.6 ± 178.6	-
Fig. 4D: Total length of central processes [µm]			
	3 months	1 year	2 years
Type I	264.7 ± 16.03	344.2 ± 20.8	370.1 ± 27.0
Type II	204.1 ± 12.8	243.9 ± 10.6	276.9 ± 23.3
Type III	233.8 ± 17.4	268.3 ± 20.5	-
Fig. 4E: Mean length of processes [µm]			
	3 months	1 year	2 years
Type I	12.7 ± 0.5	13.1 ± 0.5	15.7 ± 0.6
Type II	13.6 ± 0.7	14.1 ± 0.7	14.4 ± 0.5
Type III	14.3 ± 0.5	13.9 ± 0.6	-
Fig. 4F: Mean length of central processes [µm]			
	3 months	1 year	2 years
Type I	26.7 ± 1.6	30.3 ± 1.6	33.6 ± 1.3
Type II	29.1 ± 1.9	34.7 ± 1.7	31.0 ± 1.5
Type III	30.2 ± 1.5	32.1 ± 1.5	-

2 Supplementary Figures

2.1 Supplementary Figure 1

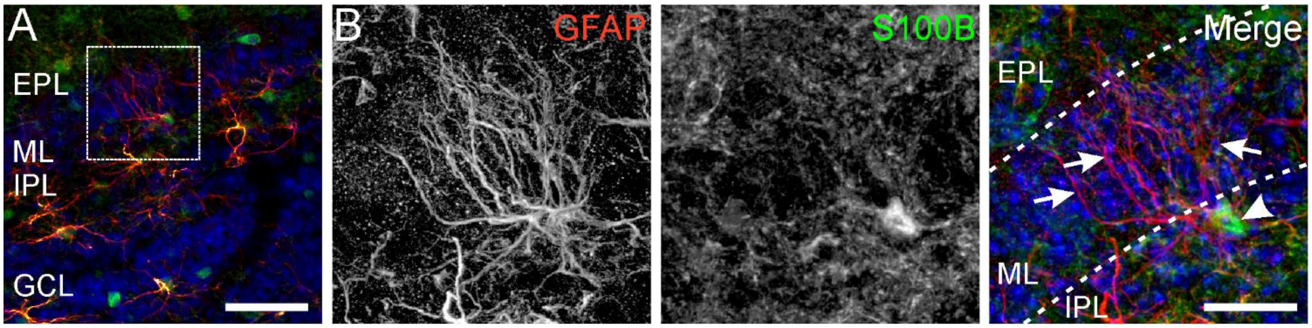

Supplementary Figure 1. A Immunostaining against GFAP (red) and S100B (green). Nuclei were stained with Hoechst 33342 (blue). Scale bar, 50 µm. **B** Magnified view of a “squid-like” type III astrocyte. The soma (arrowhead) is located in the internal plexiform layer (IPL), extending its processes (arrows) into the mitral cell layer (ML). Scale bar, 20 µm. EPL, external plexiform layer; GCL, granule cell layer.

2.2 Supplementary Figure 2

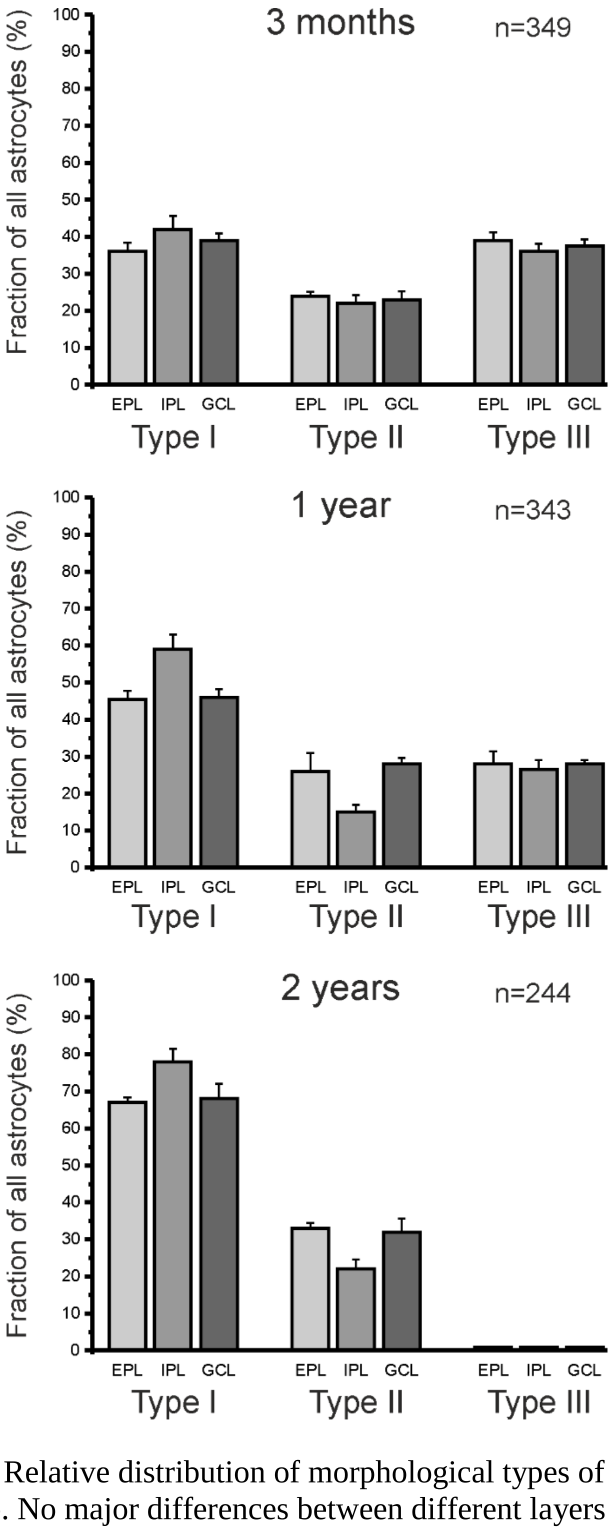

Supplementary Figure 2. Relative distribution of morphological types of astrocytes in different layers of the olfactory bulb. No major differences between different layers were found. EPL, external plexiform layer; IPL, internal plexiform layer; GCL, granule cell layer.
